# The effect of horticultural therapy on depressive symptoms among the elderly: A systematic review and meta-analysis

**DOI:** 10.3389/fpubh.2022.953363

**Published:** 2022-08-24

**Authors:** Ya Wei Zhang, Jun Wang, Tian Hong Fang

**Affiliations:** School of Art Design and Media, East China University of Science of Technology, Shanghai, China

**Keywords:** horticultural therapy, the elderly, systematic review, meta-analysis, depressive symptoms

## Abstract

**Objective:**

This systematic review and meta-analysis aimed to assess the effectiveness of horticultural therapy on depressive symptoms in the elderly and determine the potential moderators of the intervention effect.

**Methods:**

In early June 2022, randomized controlled trials and Quasi-experimental studies were searched on Web of Science, PsycINFO, CINAHL, EMBASE, Medline, PubMed, CNKI, WANFANG DATA, and CQVIP. Three independent authors proposed the following inclusion criterion for this study: the elderly with applied horticultural therapy intervention compared to non-HT intervention. From a total of 3,068 records, only 34 studies met the inclusion criteria. After the full-text screening, 13 studies were included in the analysis. An assessment of the risk of bias was conducted using RoBINS-I and RoB 2 tools. The comprehensive Meta-Analysis 3.3 tool was used for the meta-analysis.

**Results:**

Meta-analysis suggested that mean depression scores of elderly people who underwent horticultural therapy intervention were significantly lower than those who did not receive HT therapy. More significant effects were found for the elderly with mean age equal to or over 75 years instead of younger than 75 years, in randomized controlled trials instead of quasi-experimental studies, for studies with more than 20 participants receiving horticultural therapy at the same time and place instead of equal to or fewer than 20 horticultural therapy participants.

**Conclusions:**

This evidence supported that horticultural therapy had a significant positive effect on the depressive symptoms outcomes for the elderly. Therefore, our data revealed that horticultural therapy could be considered as a part of therapy in depressive symptoms reduction programs. Due to the high degree of heterogeneity and the limited number of studies, a future review is warranted to determine the effects of horticultural therapy on depressive symptoms reduction in the elderly.

**Systematic review registration:**

https://www.crd.york.ac.uk/PROSPERO/display_record.php?RecordID=272464, identifier: CRD42021272464.

## Introduction

Depression has been one of the most common mental health disorders among the elderly worldwide (1, 2). Several studies have explored the prevalence of depression in the elderly around the world in recent years (3–5). As revealed by data from the Survey on Aging and Health in Europe (SHARE), which covered information on elderly people in 27 European countries in 2015, the late-life depression rate was 29% (10483/36069), with the highest prevalence in southern Europe (35%), followed by central-eastern Europe (32%), western Europe (26%) and Scandinavia (17%) (6). Data from the China Longitudinal Study on Health and Retirement (CHARLS) suggested that rates of depressive disorder in the elderly were 41.6% (2747/6609), 32.6% (2333/7158), and 35.5% (2919/8231) in 2011, 2013, and 2015, respectively (7). Studies have shown that depression among the elderly can lead to a decrease in quality and satisfaction of life and an increase in health care costs (8, 9). Furthermore, depression has a high recurrence rate, and the elderly are particularly vulnerable to long-term harm. Research has shown that patients who have been successfully treated for depression are more than three times as likely to suffer from depression in the future as the general population (2). Therefore, even elderly people, who have overcome depression, are susceptible to relapse. Moreover, it should be noted that, after a second relapse, patients are particularly vulnerable to a longer course of depression, and after a third episode, depression is likely to become a lifetime illness, causing people to suffer from it for an extended period of time (10). As the global elderly population is expected to increase to 1.4 billion by 2030 and 2.1 billion by 2050, the global burden of depression in this age group will continue to increase (11, 12). Therefore, scholars and policymakers around the world are trying to find a solution to the depression crisis. Pharmacological treatment is the most common method of treating depression, however, continued use of these medications can result in certain side effects (13, 14). Thus, researchers have been exploring non-pharmacological treatment programs to alleviate depressive symptoms in the elderly (15). Non-pharmacological therapies, such as music therapy (16), mindfulness-based cognitive therapy (17) and animal-assisted therapy (18), have been shown to reduce depressive symptoms among elderly people. As one of the non-pharmacological therapies, horticultural therapy (HT) has been receiving increasing attention from researchers in recent decades (19). HT differs from the previously non-pharmacological therapies by encouraging human-plant interaction (20, 21).

Referring to the systematic review study by Nicholas et al. (22), the term horticultural therapy is defined as an open program that uses horticulture-based activities, whether facilitated by registered horticultural therapists or not, which is used to improve a variety of outcomes without being limited to meet specific therapeutic or rehabilitative goals. However, unlike the study from Nicholas et al., merely viewing or visiting green spaces without horticultural activities (such as planting, taking care of plants, or creating plant-related crafts) was not regarded as HT in this systematic review (23) since mere exposure to green spaces was not enough to satisfy horticulture-based activities and therefore, might not be qualified as HT intervention.

Regardless of the terminology used, researchers have conducted studies on the mechanisms of how HT promotes mental health support and reduces depressive symptoms. The biophilic tendency of humans to interact with plants and nature is one of the mechanisms linking HT to depressive symptoms (24). According to the Attention Restoration Theory (ART), HT program activities involve being in touch with plants and nature to divert attention away from negative emotions and reduce depressive feelings (25). These leisure activities in a natural setting create a sense of separation from everyday life and urban environments, which is crucial to improving mental health and reducing mental fatigue (26). HT enhances decision-making and promotes a sense of personal control and empowerment, which serves as a protective buffer from negative mental health impacts (25). A previous study of the psychophysiological relaxing effects of horticulture activities on humans also found that viewing foliage plants reduced the activity of the prefrontal cortex, increased the activity of parasympathetic nervous, improved emotional state, and reduced negative emotions compared to participants not exposed to foliage plants (27, 28). Additionally, physical activities, social cohesion, and connectedness are important mechanisms that link HT and depressive symptoms (29–31). Depressed individuals tend to spend much time in inactive behavior. One way to address this problem is to systematically change behavior by allocating more time to activities that bring fulfillment and pleasure but less time to passive or unrewarding activities. When people reduce passive activities in favor of active and beneficial behaviors, such as horticultural activities, they typically feel more fulfilled and happier, which may lead to a reduction in depressive symptoms (32). Also, in comparison with normal mental health counterparts, depressed people are significantly less satisfied with their physical health condition (33). Horticultural therapy programs that involved low-to moderate-intensity gardening activities could improve the physical function of participants (34, 35) and alleviate depressive symptoms caused by physical health concerns (36). Moreover, one of the core objectives of HT is to promote social connectedness and integration among the participants (37). According to Noone et al. (38) and Domènech-Abella et al. (39), community gardening was a way to improve social connectedness and foster community integration through its features, thus, reducing depressive symptoms caused by loneliness and lack of social interaction.

As a result of its biophilic and physical nature, its ability to restore depleted attention and encourage social connectedness. HT can help reduce depressive symptoms among the elderly; however, there is a lack of a comprehensive synthesis of the evidence on the effects of HT on reducing depressive symptoms among the elderly. Taking the two most recent systematic reviews of HT as examples, in Tu et al. (40), on the effect of HT on mental health outcomes, only two of the 18 trials included in his analysis reported the effect of HT on depressive symptoms among the elderly; in Nicholas et al. (22), on the effectiveness of HT in the elderly, only six of the 20 trials reported the effect of HT on depressive symptoms among the elderly. Additionally, since previous evidence synthesized combined measures of depressive symptoms with other constructs and did not explore the direct association between horticultural therapy and the reduction of depressive symptoms among the elderly, it was difficult to directly discern whether HT was an effective way of reducing depressive symptoms among the elderly. Such knowledge gaps limit the use of HT by those who might be willing to employ it to reduce depressive symptoms among the elderly. To address the knowledge gap, we conducted a systematic review and meta-analysis to answer whether there is sufficient evidence to support the implementation of HT as an effective intervention to reduce depressive symptoms among the elderly. Given the high rate of recurrence of depression in the elderly, prevention and treatment of depression are equally important. Thus, in this review, depressive symptoms were referred to as the various symptoms and manifestations that characterize depression rather than clinically diagnosed depression.

In our review of the literature, three novel contributions are presented. First, as far as the authors are concerned, this is the first review to focus exclusively on the effects of HT on depressive symptoms among the elderly. Second, more original and quantitative studies reporting outcomes of depressive symptoms among the elderly than previous studies were included, providing estimates of the effect of HT on depressive symptoms among the elderly. Third, in this study, the potential moderators of the intervention effects were discussed.

## Materials

Articles were searched in English and Chinese databases in early June 2022 based on the Preferred Reporting Items for Systematic Reviews and Meta-Analyses (PRISMA) statement. Kunpeuk et al. (41) and Patel et al. (42) were referenced to acquire the search terms. Nine electronic databases, i.e., Web of Science, PsycINFO, CINAHL, PubMed, Medline, EMBASE, CNKI, WANFANG DATA, and CQVIP, related to health care, psychological research, and social science, were explored regardless of the year of publication. CNKI, WANFANG DATA, and CQVIP are the most extensively used Chinese language databases. Search terms with identical English meanings were used in those Chinese databases. To match search engines, search strategies for each database were slightly inconsistent. However, the use of the main keywords was consistent (Supplementary Tables S1–S10). In addition, a manual search was conducted by selecting seemingly relevant articles from the references of the available reviews to reduce the risk of missing relevant studies (Supplementary Table S11). Y.W. and T.H. involved in the search process. The review was registered with the International prospective register of systematic reviews (PROSPERO; registration no. CRD42021272464).

### Inclusion and exclusion criteria

The inclusion criteria of this study are presented below. (i) Studies that measured depressive symptoms were included. (ii) Studies reporting original quantitative research measuring the effects of HT on depressive symptoms among the elderly. RCTs and quasi-experimental studies comparing HT interventions (as ‘treatment') with non-HT interventions or alternative interventions (as ‘control') were included. Quasi-experimental studies can also yield accurate answers. The inclusion of quasi-experimental studies can increase the external validity of results and the statistical power of a meta-analysis (43). (iii) Participants were the elderly aged 60 and above. (iv) Studies published in English or Chinese that have been peer-reviewed. Those studies which did not meet the inclusion criteria (e.g., irrelevant outcomes and referring to studies that measured some outcomes unrelated to depressive symptoms) were excluded from this study.

### Study selection process

YWZ and THF initiated the study selection process by screening the articles based on the titles and abstracts. The criteria mentioned above were followed during the screening. After the initial screen, YWZ and THF retrieved and reviewed the articles and listed the reasons for exclusion in Supplementary Table S12. The three authors resolved any disagreement in the screening through discussion. ENDNOTE X9 software was adopted to store and process the identified articles. Duplicated articles were removed. Library databases provided by East China University of Science and Technology were employed to retrieve the full text of the identified articles.

### Critical appraisal and quality assessment

The quality of the RCTs included in the meta-analysis was assessed using the RoB2 (Version 2 of the Cochrane risk-of-bias tool for randomized trials) tool (44), and the quality of quasi-experimental studies included in the meta-analysis was assessed using the ROBINS-I (Risk of Bias In Non-randomized Studies of Interventions) tool (45). YWZ and THF assessed the quality of each study independently and then cross-checked the information. All domains were classified as low risk, some concern, and high risk of bias (Supplementary Table S12–S13).

### Data extraction and analysis

YWZ and THF independently extracted study characteristics and treatment outcomes. Any disagreement was resolved by discussion until a consensus was reached or by consulting the third writer. Narrative summaries of specific findings are presented in Supplementary Table 13.

A meta-analysis was performed using Comprehensive Meta-analysis 3.3 (46). A random effects model was used to generalize the results beyond the included articles, assuming that the selected articles originated from random samples of a larger population. Effect sizes of Hedges'*g*, a Cohen variation and correct for biases due to small sample sizes, were determined (47). The magnitude of Hedge's *g* could be interpreted according to Cohen's convention, with 0.2, 0.5, and 0.8 considered small, medium, and large effect sizes, respectively (48). The degree of heterogeneity within the articles was calculated using the I2-statistic, with 0.25, 0.5, and 0.75 considered low, moderate, and high degrees of heterogeneity, respectively (49). Furthermore, due to the possibility that the results of a meta-analysis may be influenced by bias in the selection of studies, called publication bias, which can often result in an overestimation of the average effect size of an intervention, the funnel plot, and Egger's test were used to assess the risk of publication bias. A meta-analysis is unlikely to have publication bias if the selected studies are largely concentrated at the top of the funnel plot, with few at the bottom, and are equally distributed on both sides with largely symmetrical trends (50). Egger's test is based primarily on the *P*-value of the bias coefficient, and it is generally accepted that *p* > 0.05 (two-tailed) indicates that there is no publication bias (50, 51). The overall calculation was conducted by YWZ.

## Results

**Figure 1:**
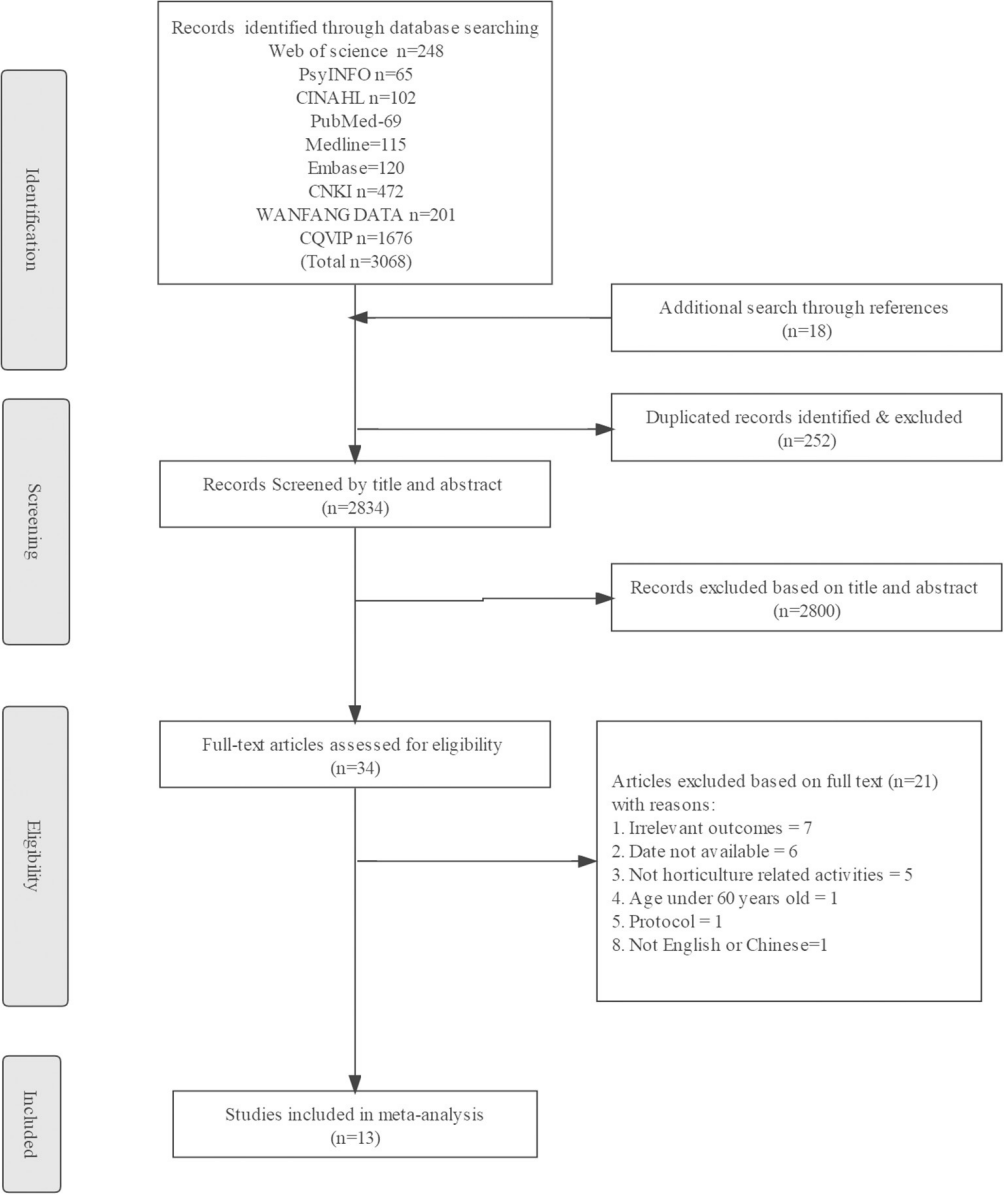
Flow diagram of the literature search and selection process.

Figure 1 presents the overall article selection criteria. On the whole, 3,068 articles from electronic searches and 18 articles from manual searches were covered. After deleting duplicates, 2,800 studies were used for the screening of the title and summary. Of the 2,800 articles retrieved, 34 were read in full. Lastly, 13 articles were selected for the review, while 21 were excluded. Reasons for exclusion were listed in Supplementary Table S12.

### Location

Study locations of the 13 articles were reported in only two continents, Asia and Oceania. The majority of the studies were conducted in Asia (*n* = 12), among which five articles were reported in China (52–56), four in Japan (57–60), two in South Korea (61, 62), and one in Singapore (63). The study conducted in Oceania was in Australia (64).

### Population and sample size

Most articles (*n* = 8/13) indicated that their participants were recruited from nursing homes (52, 53, 55, 58, 59, 61, 63, 64), two from neighborhoods (57, 60), two from hospitals (54, 56), and one from a homeless living facility (62). Some studies were conducted on specific populations. These included participants with significant depressive symptoms (55–57), dementia (64), and post-traumatic stress disorder (60). In three studies, all participants were female (55, 60, 61). Nine studies had both male and female participants. One study did not describe the gender of the participants (59).

There were 608 subjects involved in the 13 studies, with sample sizes ranging from 6 to 150. For sampling methods and data collection, all 13 articles employed convenience sampling and undertook a self-reported survey.

Except for one article that only stated that people over 60 years were recruited without explicitly giving the mean age (54). The mean sample age of the remaining studies ranged from 65.15 to 90.3 years. In previous studies, a cutoff age of 75 years was used as the separating age between young-old (< 75 years) and old-old (≥ 75 years) (65–68). Based on the above separation criteria, seven studies focused on old-old (52, 53, 58, 59, 61, 63, 64), and five studies focused on young-old (55–57, 60, 62).

### Study design

Among the 13 articles included, seven were identified as quasi-experimental studies (53, 55, 58, 61–64), and six were identified as RCTs (52, 54, 56, 57, 59, 60). For RCTs, two applied single-blind RCT (57, 60), and one of which is crossover RCT (60).

### Intervention and control

The contents of HT interventions and control interventions were different in different articles. Besides gardening activities (e.g., seeding, cultivating, and harvesting) performed by nearly all subjects, the HT interventions mentioned in the articles also included nature-art activities [e.g., flower arrangement (53, 54, 56, 59, 61, 62), decorating drawing papers with dry flowers and leaves (52, 55, 63), dyeing nails with garden balsam (62)], nutrition education (57), field trips to green land (55, 62, 64), and cooking and tasting (55, 58, 62). Additionally, of the 13 studies, eight were facilitated by registered horticultural therapists (52, 55, 60–63) or professionals trained in horticultural techniques (58, 59), while five studies did not indicate whether registered horticultural therapists or professionals trained in horticultural techniques were involved (53, 54, 56, 57, 64). For the controls, two studies arranged extra interventions besides routine activities. Yuka et al. (60) arranged a weekly 60-min session consisting of a lecture on stress education for the control group. Zhen Lan et al. (56) reported that an irregularly arranged 30-min sports nursing program was included in the control intervention.

Regarding the duration of the intervention session in the HT groups, the shortest session duration was 250 seconds (59), six studies conducted a 60–90 min intervention session (53, 55, 57, 60, 62, 63), and the longest session duration was 120 min (52, 54, 56). For the HT sessions lasting only 250 seconds, a simple indoor HT program called “Bedside structured floral arrangement (SFA)” was used. This program was developed specifically for the elderly, who required assistance with their mobility (59). In terms of the frequency of intervention, the intervention was conducted once a week in nine studies (52, 53, 55, 57–60, 62, 63), twice a week in one study (61), and six times a week in one study (56). Two studies did not describe the number of interventions per week (54, 64). In terms of the length of the intervention, the shortest duration was 1 day (59), and the longest durations in two different studies were 1 (56) and 2 years (54). Ten studies conducted an intervention for 6–24 weeks, with 6 weeks (*n* = 2) and 8 weeks (*n* = 3) as the most widely used length of intervention.

### Depressive symptoms measurement tools

Different depressive symptoms measurement tools were applied. Geriatric Depression Scale (GDS) was the most widely used, appearing in nine studies, with six studies using the short version of GDS (GDS-15) (52, 53, 55, 57, 58, 60), and two studies using the Korean version of GDS-15 (61, 62). Other tools applied consisted of Self-Rating Depression Scales (SDS) (54, 63) and Cornell Scale for Depression in Dementia (CSDD) (59, 64).

### Risk of bias and quality of the selected articles

The results of the assessment of risk of bias are listed in Supplementary Tables S12, S13. The RoB2 tool was used to evaluate six randomized studies, and the ROBINS-I tool was used to evaluate seven quasi-experimental studies. All randomized studies followed an acceptable randomization process, deviations from intended interventions, measurement of the results, and selection of the reported results. Three studies (54, 56, 60) were classified as high risk for not granting the dropout rate of participants. All quasi-experimental studies followed acceptable confounders, classification of interventions, deviations from intended interventions, missing data, measurement of the results, and selection of the reported results. A study (64) was classified as high risk because it selected participants based on the physical and mental health of the individuals observed after the start of the intervention, and two participants with significantly decreased physical and mental health were excluded from this study.

### Narrative summary

As reported in most articles (*n* = 9/13), the elderly exhibited significantly lower levels of depression after receiving HT interventions. Among the nine articles, one proposed a follow-up evaluation, and the result suggested that the reduction effect of depressive symptoms of the HT intervention was maintained even 8 weeks after the sessions ended (60). However, for control groups without active interventions, none showed a significant decrease in depression scores after the trials. For control groups receiving other active interventions (*n* = 2/13), one (60) had a significant increase in GDS scores after receiving a stress control education intervention, while the HT group had a significant decrease in GDS scores, and the other (56) had a significant decrease in depression scores after receiving sports nursing interventions, although the effect was weaker than that of the HT intervention used in this study.

### Overall meta-analysis of HT and depressive symptoms

**Figure 2:**
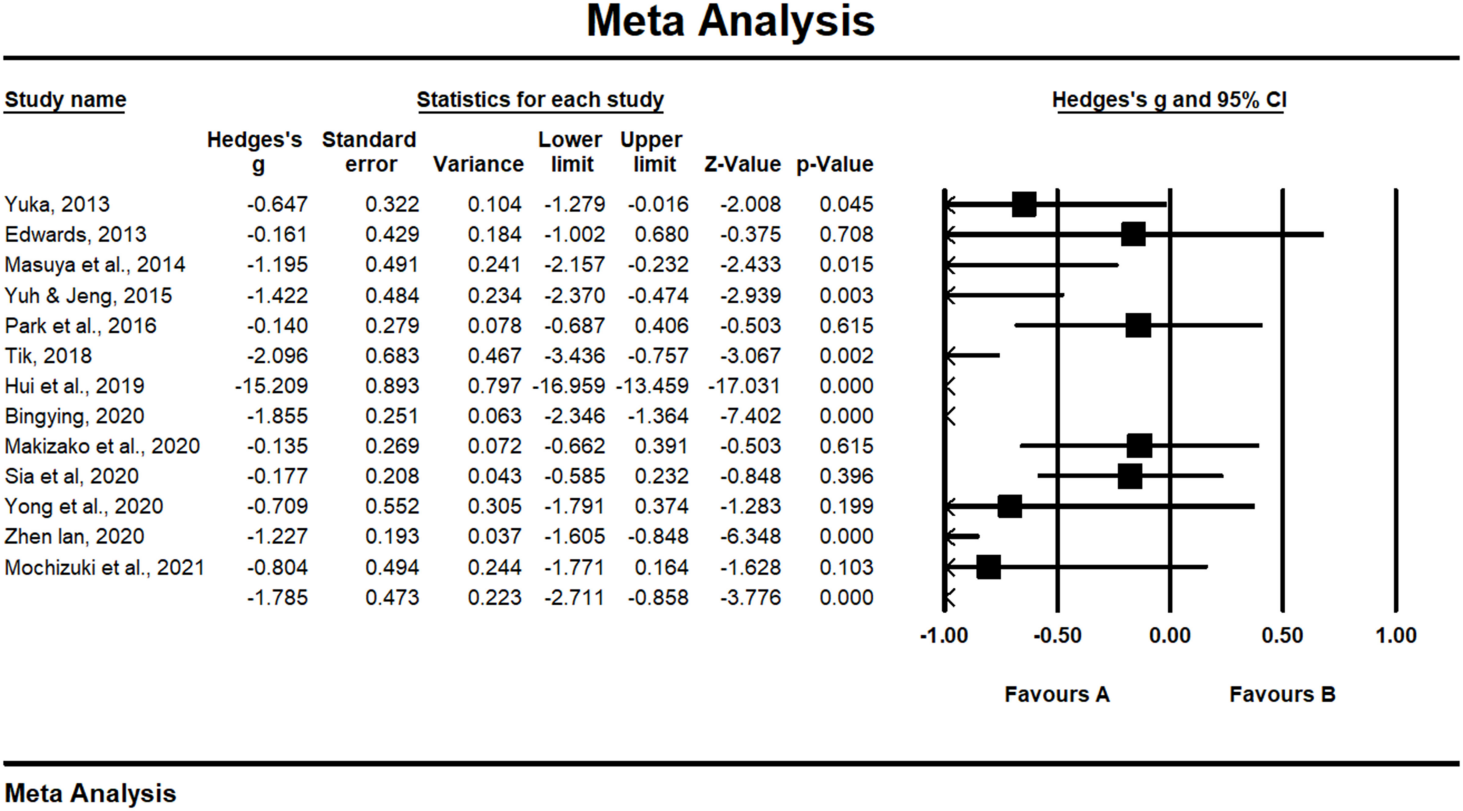
Forest plot: HT group vs. control for depression scores (continuous outcome).

Thirteen articles were included in the meta-analysis to investigate the immediate post-test effects of HT on the depressive symptoms of the elderly. We extracted the mean value, sample size (*n*), and standard deviation (SD) of depressive symptoms from the included articles. For articles measuring the outcomes of depressive symptoms during HT interventions at multiple time points, only data points at the start and end were applied. The results of the meta-analysis suggested that the mean depression scores of the elderly, who received HT interventions, were significantly lower than those without HT interventions (Hedge's g = −1.785, *p* < 0.001) (Figure 2). The I^2^ value was 96.128% (*P* < 0.001). I^2^ ≥75% with statistical significance suggested a high degree of heterogeneity between articles. After removing the four studies (54, 56, 60, 64), which were classified as high risk, the results remained stable and significant, with Hedge's g of −2.274 (*p* = 0.003) and I^2^ value of 97.205% (*P* < 0.001) (Supplementary Figure 1). After removing the two studies (56, 60) in which alternative interventions were used in the control groups, Hedge's g was −2.06 (*p* = 0.001), and the value of I^2^ was 96.737% (*P* < 0.001) (Supplementary Figure 2). These results suggested that HT had a significant positive effect on the reduction of depressive symptoms among the elderly, regardless of whether alternative interventions were used in the control groups.

### Subgroup meta-analysis

Subgroup analysis was conducted to assess the source of heterogeneity. Each (categorical) subgroup variable should have at least four studies, and this number is the lower bound for considering a subgroup analysis (69). These articles were divided into two subgroups based on age group (young-old, *n* = 5; old–old, *n* = 7), study design (RCTs = 6, Quasi-experimental studies = 7), and the number of participants who received the HT intervention at the same time and the same place in each study (under or equal to 20 = 8; above 20 = 5). The value between groups of the Q-statistic was used to statistically assess heterogeneity. Since the Q statistic has a low differential power, a value of *P* < 0.1 is considered to indicate significant differences between groups (70, 71). The results indicated that there were significant differences in the effect size (Hedge's *g*) of variables on the outcome of depressive symptoms in the two subgroups of all three types. The effect of the intervention on the old-old was significantly greater than that on the young-old (*g*: 2.574 > 0.855, *p* = 0.098 < 0.1). The intervention effect of the RCTs was significantly greater than that of the quasi-experimental studies (*g*: 3.070 > 0.679, *p* = 0.013 < 0.1). Moreover, the intervention effect of programs with more than 20 HT participants was significantly greater than those with fewer HT participants (*g*: 3.434 > 0.760, *p* = 0.013 < 0.1) (Table 1).

**Table 1:** Summary of the meta-analysis for six subgroups.

**Subgroup**	**No. of comparison**	**Effect size**		**Heterogeneity**	
		**Hedge's *g***	***P*-value**	***Q*-value**	***P*-value**
Age group					
Young-old	5	−0.855	0.010	2.744	0.098 <0.1
Old-old	7	−2.574	0.004		
Study design					
RCT	6	−0.3070	0.001	6.208	0.013 <0.1
Quasi-experimental studies	7	−0.679	0.006		
Participants' number					
Under or equal to 20	8	−0.760	0.000	6.160	0.013 <0.1
Above 20	5	−3.434	0.001		

### Results of publication bias

**Figure 3:**
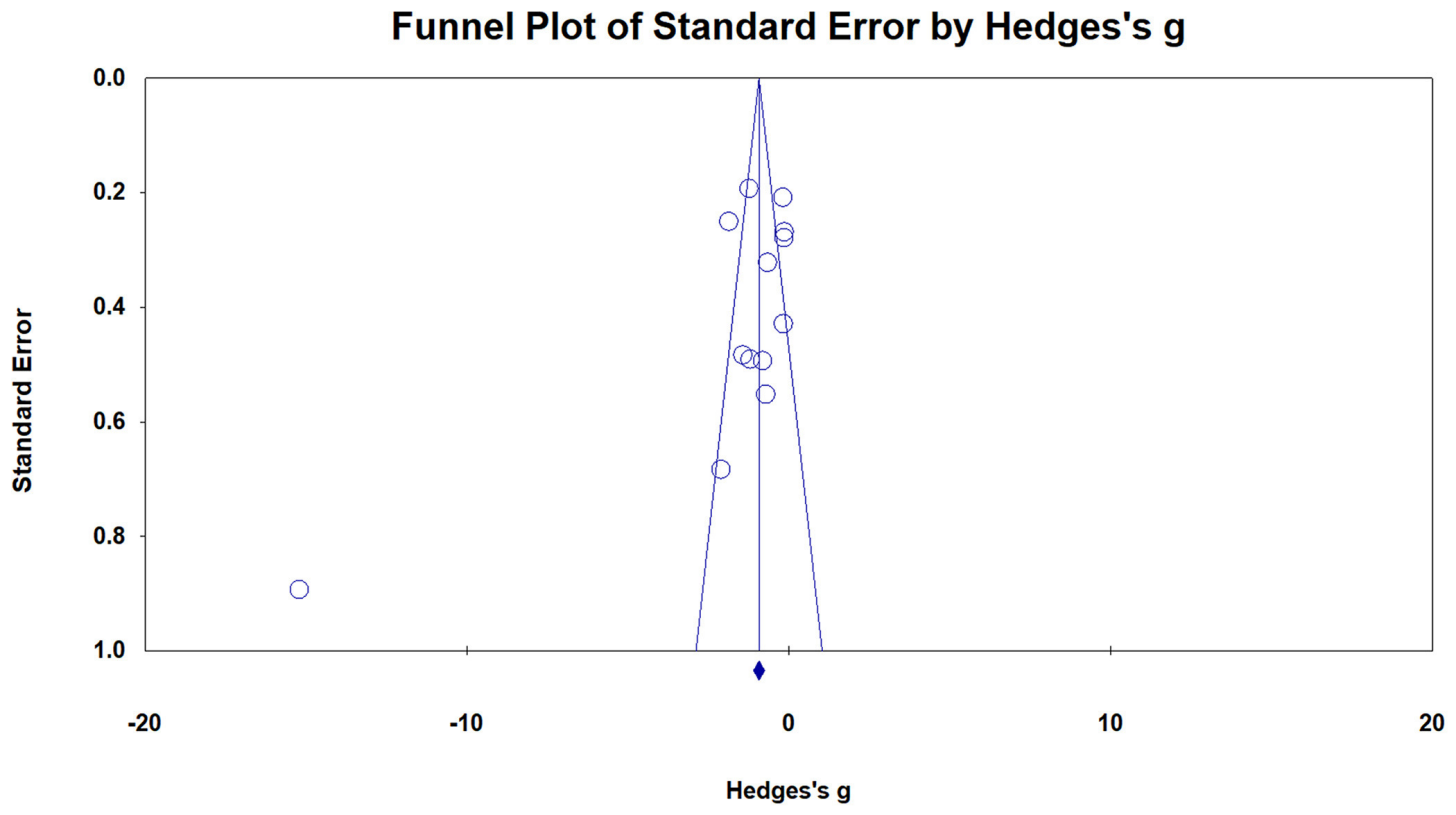
Funnel plot of articles on HT intervention and depression scores in the elderly.

The funnel plot shows an approximate symmetrical distribution of study effect size, which suggests that there might not be any publication bias (Figure 3). Egger's test further verified the conclusion. The bias coefficient of Egger's test was 0.11326 (*p*-value >0.05, two-tailed), so the evidence of publication bias is weak.

## Discussion

As a result of this study, we found that HT could significantly reduce depressive symptoms. It can be concluded that HT is effective in reducing depressive symptoms in the elderly. Horticultural activities, such as watering, weeding, etc., can enable the elderly to move their limbs, exercise their entire body, promote the recovery of body functions, increase physical fitness, promote metabolism, and reduce depressive emotions caused by concerns about physical health (61). The elderly can feel relaxed, relieve stress, and reduce negative emotions by paying attention to changes in plants, rubbing parts of plants with their own hands, and smelling natural scents emitted by plants (54). Taking care of plants and sharing experiences with others give the elderly a sense of satisfaction, accomplishment, and self-confidence (52). Elderly people who experience a sense of satisfaction, accomplishment, and self-confidence are less likely to have depression symptoms (53). Additionally, activities of the HT program help the elderly to expand their social networks, become friends with members of the group, and help them feel accepted, which enables them to live a more active life and reduce feelings of depression (55).

Compared with the young-old, positive effects of HT were particularly evident in the old-old. Positive activities and social interactions for the old-old are difficult to develop spontaneously due to their physical conditions having declined more significantly than those of the young-old (72, 73). A limited amount of exercise and social interaction would cause a further decline in physical and mental health. Using horticulture as a medium, the activities offer the old-old to have the opportunity to increase positive physical activities. Taking care of plants allows the old-old to gain a sense of achievement and pleasure, as well as improve physical health conditions (74), thus reducing feelings of depression caused by lack of achievement and concerns about physical health decline (75). Furthermore, participants often interact actively and happily with each other during horticultural activities (53). Horticulture, as a topic of mutual interest, facilitates conversation, improves social integration, promotes mental health, and reduces depressive symptoms in old-old (76, 77).

The present meta-analysis suggested that studies using RCTs found significantly larger effects compared to quasi-experimental studies. The results of RCTs can better rule out alternative explanations for established intervention effects than non-randomized designs. Selection bias in studies of nonrandom effects often leads to an overestimation of the effectiveness of treatment (78), but this did not occur in the present study.

More significant effects were found in studies with more than 20 HT participants compared to those with 20 or fewer HT participants. Social connectedness is extremely important for better mental health, and people with fewer interpersonal relationships or lower levels of social support have consistently higher rates of depression (79). HT intervention programs with a larger number of participants could provide the elderly with more opportunities to meet more people, expand their social connectedness, and increase interpersonal relationships. This may account for the greater effectiveness of HT interventions in reducing depressive symptoms with a higher number of participants.

Despite a favorable outcome of HT in the reduction of depressive symptoms among the elderly in this study, it is worthwhile to be aware of some methodological caveats. In terms of research location, included studies were only conducted in Asia and Oceania. No study was conducted on other continents, such as Europe. This could be a key confounding factor for the accuracy of the review results (80). For the sampling method, convenience sampling was applied in all included articles, thus, suggesting weak external validity. Moreover, non-response proportion was not reported in most articles, which could lead to non-response bias. In addition, all articles used self-reported surveys for data collection, which may result in systematic errors. Moreover, some articles are at a high risk of bias based on the results of quality assessment, and this could lead to internal validity bias.

It is acknowledged that this review has some weaknesses. First, non-English and non-Chinese evidence was not included in the review. Second, qualitative studies, which were not included, could explain the mechanism of HT in more detail. Third, due to the limited number of studies enrolled, some relationships between HT and depressive symptoms reduction, such as the connection between HT intensity and depressive symptoms, the comparison between the effects of HT on the first and recurrent depression, or the comparison of the effects of HT on elderly individuals with a serious mental or physical disability with those in good health, were not subgroup analyzed.

Subsequent research is recommended. For more effective measurement, novel biological detection technologies, e.g., electroencephalogram testing (81) or blood examination (82), are recommended because they can provide a more objective indication of depression levels than self-reported methods. Additionally, more evidence is required from other continents. Furthermore, more research is necessary for all age groups to better understand the causal relationship between HT and depressive symptoms.

## Conclusion

In summary, HT has a significant positive effect on reducing depressive symptoms in the elderly. Subgroup analysis suggested that HT had a more positive effect on the old-old. Programs with more than 20 HT participants at the same time and in the same place were more effective. However, the number of original studies included in this meta-analysis was limited, and more articles are required to identify the causal correlation between HT and reduction of depressive symptoms among the elderly and the evaluation of challenges, such as disadvantages and advantages of the promotion of HT is popularly applied in the elderly.

## Data availability statement

The original contributions presented in the study are included in the article/Supplementary material, further inquiries can be directed to the corresponding author.

## Author contributions

Study design and writing—review and editing: YWZ and JW. Methodology: YWZ and THF. Formal analysis and writing—drafting: YWZ, JW, and THF. All authors contributed to the article and approved the submitted version.

## Conflict of interest

The authors declare that the research was conducted in the absence of any commercial or financial relationships that could be construed as a potential conflict of interest.

## Publisher's note

All claims expressed in this article are solely those of the authors and do not necessarily represent those of their affiliated organizations, or those of the publisher, the editors and the reviewers. Any product that may be evaluated in this article, or claim that may be made by its manufacturer, is not guaranteed or endorsed by the publisher.
