# Supplementary material for: The effect of horticultural therapy on depressive symptoms among the elderly: A systematic review and meta-analysis
Source: Front Public Health. 2022 Aug 24;10:953363. doi: 10.3389/fpubh.2022.953363 (PMC9449424; doi:10.3389/fpubh.2022.953363)

**Supplementary Table 1**

Search strategy

| **Domains** | **Search terms** |
| --- | --- |
| Gardening | gardening、horticulture、horticultural activit*、horticultural therapy |
| older people | Aged, Aged 60 and over, Older adult*, Senior*, Elder*, Geriatric*, Aging, Old people, Aging adult*, Old men, Old women, Older people, Older man, Older woman, Older person |
| Depression | depress*, mental |

**Supplementary Table 2**

Web of Science (Core Collection) was searched on 06/06/22

|  | **Search terms** | **Hits** |
| --- | --- | --- |
| #1 | TS=(Garden* OR Horticultur*) | 81,629 |
| #2 | TS=(aged OR Older adult* OR Senior* OR Elder* OR Geriatric* OR Aging OR Old people OR Aging adult* OR Old men OR Old women OR Older people OR Older man OR Older woman OR Older person) | 4,382,850 |
| #3 | #1 AND #2 | 4,840 |
| #4 | TS=(depress* OR mental) | 1,198,934 |
| #5 | #3 AND #4 | 318 |
| #6 | #5  Refined by: Articles (Document Types) | 260 |
| #7 | #5  Refined by: Articles (Document Types) and English or Chinese (Languages) | 248 |

**Supplementary Table 3**

APA PsycINFO was searched using on 06/06/22

|  | **Search terms** | **Hits** |
| --- | --- | --- |
| #1 | AB Horticultur* or Garden* | 3,208 |
| #2 | TI Horticultur* or Garden* | 1,052 |
| #3 | #1 OR #2 | 3,468 |
| #4 | AB (Aged OR Aged 60 and over OR Older adult* OR Senior* OR Elder* OR Geriatric* OR Aging OR Old people OR Aging adult* OR Old men OR Old women OR Older people OR Older man OR Older woman OR Older person) | 444,731 |
| #5 | TI (Aged OR Aged 60 and over OR Older adult* OR Senior* OR Elder* OR Geriatric* OR Aging OR Old people OR Aging adult* OR Old men OR Old women OR Older people OR Older man OR Older woman OR Older person) | 111,285 |
| #6 | #4 OR #5 | 458,246 |
| #7 | #3 AND #6 | 408 |
| #8 | T1(depress* OR mental) | 246,012 |
| #9 | AB(depress* OR mental) | 672,484 |
| #10 | #8 OR #9 | 696,003 |
| #11 | #7 AND #10 | 68 |
| #12 | #11  Narrow by Language: - English | 65 |

**Supplementary Table 4**

CINAHL was searched using the EBSCOhost on 06/06/22

|  | **Search terms** | **Hits** |
| --- | --- | --- |
| #1 | MH "Horticulture" | 3,330 |
| #2 | TI Horticultur* OR Garden* | 2,219 |
| #3 | AB Horticultur* OR Garden* | 3,283 |
| #4 | #1 OR #2 OR #3 | 6,347 |
| #5 | MH "Aged+" | 915,669 |
| #6 | AB Aged OR Aged 60 and over OR Older adult* OR Senior* OR Elder* OR Geriatric* OR Aging OR Old people OR Aging adult* OR Old men OR Old women OR Older people OR Older man OR Older woman OR Older person | 404,992 |
| #7 | #5 OR #6 | 1,136,659 |
| #8 | #4 AND #7 | 912 |
| #9 | MH "Depression" | 121,877 |
| #10 | TI depress* OR mental | 146,011 |
| #11 | AB depress* OR mental | 286,512 |
| #12 | #9 OR #10 OR #11 | 363,159 |
| #13 | #8 AND #12 | 106 |
| #14 | #13  Narrow by Language: - English | 102 |

**Supplementary Table 5**

PubMed was searched using the EBSCOhost on 06/06/22

|  | **Search terms** | | **Hits** | |
| --- | --- | --- | --- | --- |
| #1 | All Fields Horticultur* OR Garden* | | 74,325 | |
| #2 | All Fields Aged OR Aged 60 and over OR Older adult* OR Senior* OR Elder* OR Geriatric* OR Aging OR Old people OR Aging adult* OR Old men OR Old women OR Older people OR Older man OR Older woman OR Older person | | 6,827,537 | |
| #3 | All Fields depress* OR mental | | 1,240,894 | |
| #4 | #1 AND #2 AND #3 | | 638 | |
| #5 | | #4  Refined by: Books and Document, Clinical Trial and Randomized Controlled Trial (Article Type) | | 69 |

**Supplementary Table 6**

Medline was searched on 06/06/22

|  | **Search terms** | | | **Hits** |
| --- | --- | --- | --- | --- |
| #1 | AB =( Horticultur* OR Garden**)* | | | 19,945 |
| #2 | AB=( Aged OR Aged 60 and over OR Older adult* OR Senior* OR Elder* OR Geriatric* OR Aging OR Old people OR Aging adult* OR Old men OR Old women OR Older people OR Older man OR Older woman OR Older person) | | | 1,504,088 |
| #3 | AB=( depress* OR mental) | | | 1,051,072 |
| #4 | #1 AND #2 AND #3 | | | 159 |
| #5 | | #4  Narrow by Language: - English | 153 | |
| #6 | | #5  Exact duplicates removed from the results. | 115 | |

**Supplementary Table 7**

EMBASE was searched on 06/06/22

|  | **Search terms** | **Hits** |
| --- | --- | --- |
| #1 | ti,ab,kw Horticultur* OR Garden* | 20,997 |
| #2 | ti,ab,kw Aged OR Aged 60 and over OR Older adult* OR Senior* OR Elder* OR Geriatric* OR Aging OR Old people OR Aging adult* OR Old men OR Old women OR Older people OR Older man OR Older woman OR Older person | 1,015,959 |
| #3 | ti,ab,kw depress* OR mental | 1,153,065 |
| #4 | #1 AND #2 AND #3 | 120 |

**Supplementary Table 8**

CNKI was searched on 06/06/22

|  | **Search terms** | **Hits** |
| --- | --- | --- |
| #1 | 全文=园艺（模糊） | 703,726 |
| #2 | 全文=老年OR老人OR长者（模糊） | 27,967 |
| #3 | #1 AND #2 | 1687 |
| #4 | 全文=抑郁OR健康OR心理OR情绪（模糊） | 519,133 |
| #5 | #3 AND #4 | 472 |

**Supplementary Table 9**

WANFANG DATA was searched on 06/06/22

|  | **Search terms** | **Hits** |
| --- | --- | --- |
| #1 | 全部=园艺（模糊） | 37,376 |
| #2 | 全部=老年OR老人OR长者（模糊） | 1,111,912 |
| #3 | #1 AND #2 | 369 |
| #4 | 全部=抑郁OR健康OR心理OR情绪（模糊） | 5,322,965 |
| #5 | #3 AND #4 | 201 |

**Supplementary Table 10**

CQVIP was searched on 06/06/22

|  | **Search terms** | **Hits** |
| --- | --- | --- |
| #1 | 任意字段=园艺（模糊） | 1,006,806 |
| #2 | 任意字段=老年OR老人OR长者（模糊） | 968,031 |
| #3 | #1 AND #2 | 4,024 |
| #4 | 任意字段=抑郁OR健康OR心理OR情绪（模糊） | 4,546,186 |
| #5 | #3 AND #4 | 1,676 |

**Supplementary Table 11**

Related review for hand search for other sources

| **No.** | **Author** | **Objective** |
| --- | --- | --- |
| 1 | Yao, Y. F. , & Chen, K. M. . (2017). Effects of horticulture therapy on nursing home older adults in southern taiwan.*Quality of Life Research*, **26(4)**, 1007-1014. | To test the effects of horticulture therapy on activities of daily living, happiness, meaning of life, and interpersonal intimacy of nursing home older adults in southern Taiwan. |
| 2 | Tse, M. . (2010). Therapeutic effects of an indoor gardening programme for older people living in nursing homes. *Journal of Clinical Nursing*, **19(7-8)**, 949-958. | To explore the activities of daily living and psychological well-being of older people living in nursing homes and also to examine the effectiveness of a gardening programme in enhancing socilaisation and life satisfaction, reducing loneliness and promoting activities of daily living for older people living in nursing homes. |
| 3 | Han, J. W. , Han, C. , Yo-Han, J. , Chong-Hyeon, Y. , Jong-Min, W. , & Won, K. . (2016). The effects of forest therapy on coping with chronic widespread pain: physiological and psychological differences between participants in a forest therapy program and a control group. *International Journal of Environmental Research and Public Health*,**13(3)**, 1-13: | To investigate the effects of a two-day forest therapy program on individuals with chronic widespread pain. |
| 4 | Tom, Barnicle, Karen, Stoelzle, & Midden. (2003). The effects of a horticulture activity program on the psychological well-being of older people in a long-term care facility. *HortTechnology*, **13(1)**, 81-85. | To investigate the effects of indoor horticulture activities on the current psychological well-being of older people in two long-term care facilities over a 7-week period. |
| 5 | Bassi, M. , Rassiga, C. , Fumagalli, N. , & Senes, G. . (2018). Quality of experience during horticultural activities: an experience sampling pilot study among older adults living in a nursing home. *Geriatric Nursing*, **39**:457-464 | To assess the exercise intensities of nine common gardening tasks in older adults |
| 6 | Murphy, P. F. , Miyazaki, Y. , Detweiler, M. B. , & Kim, K. Y. . (2010). Longitudinal analysis of differential effects on agitation of a therapeutic wander garden for dementia patients based on ambulation ability. *Dementia*, **9(3)**, 355-373. | To assess the impact of visiting a wander garden on monthly agitation levels of a group of elderly veterans diagnosed with dementia, with attention to their ambulatory ability. |
| 7 | Hawkins, J. L. , Thirlaway, K. J. , Backx, K. , & Clayton, D. A. . (2011). Allotment gardening and other leisure activities for stress reduction and healthy aging. *Horttechnology*, **21(5)**, 577-585. | To explore the potential benefits of allotment gardening for healthy aging, focusing on the opportunities for outdoor physical activity, social support, and contact with nature that allotment gardening provides. |
| 8 | Kotozaki, Y. . (2013). The psychological changes of horticultural therapy intervention for elderly women of earthquake-related areas. *Trauma & Treatment*,**03(01)**,1-6. | To examine the psychological changes of horticultural therapy intervention for elderly women of earthquake-related areas. |
| 9 | Jarrott, S. E. , & Gigliotti, C. M. . (2010). Comparing responses to horticultural-based and traditional activities in dementia care programs. *American Journal of Alzheimer’s Disease and Other Dementias*, **25(8)**, 657-665. | To Compare responses to horticultural-based and traditional activities in dementia care programs |
| 10 | Luk K Y, Lai K Y C, Li C C, et al..(2011) The effect of horticultural activities on agitation in nursing home residents with dementia.*International Journal of Geriatric Psychiatry*,**26(4)**, 435-436. | To examine the effect of horticultural activities on agitation in nursing home residents with dementia |
| 11 | CA Edwards, Mcdonnell, C. , & Merl, H. . (2013). An evaluation of a therapeutic garden's influence on the quality of life of aged care residents with dementia. *Dementia*,**12(4)**, 494-510. | To evaluate whether a therapeutic garden can improve the quality of life of aged care residents with dementia and their carers, objective instruments as well as interviews with residents, staff, and family members were employed. |
| 12 | Masuya, J., Ota, K., & Mashida, Y. (2014). The effect of a horticultural activities program on the psychologic, physical, cognitive function and quality of life of elderly people living in nursing homes.*Int J Nurs Clin Pract*, **1(10.15344)**, 2394-4978. | To examine the effects of a horticultural activities program on the psychologic, physical, and cognitive function and quality of life in elderly residents of nursing homes. |
| 13 | Kim, Y. H., Lee, S. H., Park, C. S., Bae, H. O., Kim, Y. J., & Huh, M. R. (2020). A Horticultural Therapy Program Focusing on Gardening Activities to Promote Psychological, Emotional and Social Health of the Elderly Living in a Homeless Living Facility for a Long Time: A Pilot Study. *Journal of People, Plants, and Environment*,**23(5)**, 565-576. | To determine the psychological, emotional, and social effects of a horticultural therapy program composed of gardening activities, which was designed based on the semantic structures of life for the homeless elderly living in the facilities for a long time. |

**Supplementary Table 12**

Excluded studies with reasons

| **No** | **Authors** | **Reasons for exclusion** | **Objective** |
| --- | --- | --- | --- |
| 1 | Austin, E. N., Johnston, Y. A., & Morgan, L. L. (2006). Community gardening in a senior center: A therapeutic intervention to improve the health of older adults. *Therapeutic Recreation Journal*,**40(1)**, 48-56. | Mean and SD regarding depression were not provided | To examine what effect, if any, a community gardening activity at a senior centre might have on the level of functional health, depression, and physical fitness for independent-living elders. |
| 2 | Barnicle, T., & Midden, K. S. (2003). The effects of a horticulture activity program on the psychological well-being of older people in a long-term care facility.  *HortTechnology*,**13(1)**, 81-85. | No report on depression outcomes | To investigate the effects of indoor horticulture activities on the current psychological well- being of older people in two long- term care facilities over a 7-week period. |
| 3. | Rappe, E., & Kivelä, S. L. (2005). Effects of garden visits on long-term care residents as related to depression. *HortTechnology*, ***15*(2)**, 298-303. | No horticulture related | To investigate the perceived effects and meanings related to garden visits among older individuals living in long-term care and assessed whether there are associations between experiences from garden visits and self-rated depression. |
| 4. | Lin, T. Y., Huang, C. M., Hsu, H. P., Liao, J. Y., Cheng, V. Y. W., Wang, S. W., & Guo, J. L. (2020). Effects of a Combination of three-dimensional virtual reality and hands-on horticultural therapy on institutionalized older adults’ physical and mental health: Quasi-experimental design. *Journal of medical Internet research*, ***22*(11)**, e19002. | Mean and SD regarding depression were not provided | To explore the effects of a combination of 3D virtual reality and horticultural therapy on institutionalized older adults’ physical and mental health. |
| 5 | Chan, H. Y., Ho, R. C. M., Mahendran, R., Ng, K. S., Wai-San Tam, W., Rawtaer, I. & Kua, E. H. (2017). Effects of horticultural therapy on elderly’health: protocol of a randomized controlled trial. *BMC geriatrics*,**17(1)**, 1-10. | Protocol | To evaluate the efficacy of HT in promoting Asian elderly’ mental health, cognitive functioning and physical health. |
| 6. | Kim, M. Y., Kim, G. S., Mattson, N. S., & Kim, W. S. (2010). Effects of horticultural occupational therapy on the physical and psychological rehabilitation of patients with hemiplegia after stroke. *Horticultural Science & Technology*, *28*(5), 884-890. | Not English or Chinese | To examine the effects of horticultural occupational therapy (HOT) on the physical and psychological rehabilitation of stroke patients with paralysis on one side of the body |
| 7. | Ng, K. S. T., Sia, A., Ng, M. K., Tan, C. T., Chan, H. Y., Tan, C. H., ... & Ho, R. (2018). Effects of horticultural therapy on Asian older adults: A randomized controlled trial. International journal of environmental research and public health, 15(8), 1705. | Mean and SD regarding depression were not provided | To investigate the effectiveness of HT in improving mental well-being and modulating biomarker levels. |
| 8 | Jarrott, S. E., & Gigliotti, C. M. (2010). Comparing responses to horticultural-based and traditional activities in dementia care programs. *American Journal of Alzheimer's Disease & Other Dementias*, **25(8)**, 657-665. | No report on depression outcomes | To compare responses to horticultural-based and traditional activities in dementia care programs |
| 9 | McCaffrey, R., Hanson, C., & McCaffrey, W. (2010). Garden walking for depression: a research report. *Holistic nursing practice*,**24(5)**, 252-259. | Not horticulture related activities | To determine the effect of garden walking and reflective journaling on adults who are 65 years old and older with depression. |
| 10 | McCaffrey, R., Liehr, P., Gregersen, T., & Nishioka, R. (2011). Garden walking and art therapy for depression in older adults: a pilot study. *Research in gerontological nursing*, **4(4)**, 237-242. | Not horticulture related activities | To compare garden walking (either alone or guided) with art therapy in older adults with depression. |
| 11 | Palsdottir, A. M., Stigmar, K., Norrving, B., Petersson, I. F., Astrom, M., & Pessah-Rasmussen, H. (2020). Nature-based rehabilitation to reduce post-stroke fatigue is not effective: a randomized controlled trial. *Journal of Rehabilitation Medicine*, **52(2)**, 1-7. | Age starting from 50 years | To determine whether nature-based rehabilitation, as an add-on to standard care, has a long-term influence on post-stroke fatigue, perceived value of everyday occupations, disability, health-related quality of life, anxiety, and depression at follow-up 8 and 14 months after randomization. |
| 12 | Szczepańska-Gieracha, J., Cieślik, B., Serweta, A., & Klajs, K. (2021). Virtual Therapeutic Garden: A Promising Method Supporting the Treatment of Depressive Symptoms in Late-Life: A Randomized Pilot Study. *Journal of Clinical Medicine*, **10(1942)**, 1-13. | Not horticulture related activities | To evaluate the effectiveness of virtual therapy in the elderly for whom the previous multimodal, biopsychosocial therapeutic programme had not brought the expected results. |
| 13 | Wen, Y., Liu, L., Xun, Z., Jiaying, M., Shuyi, C., Wanwen, H., ... & Wang, Z. (2020). Effect of a Rehabilitation Garden on Rehabilitation Efficacy in Elderly Patients with Chronic Obstructive Pulmonary Disease. *Pakistan Journal of Zoology*, *52*(6), 2393. | Not horticulture related activities | To evaluate the efficacy of an outdoor rehabilitation program performed in a garden setting for elderly patients affected with chronic obstructive pulmonary disease |
| 14 | Lai, C. K., Kwan, R. Y., Lo, S. K., Fung, C. Y., Lau, J. K., & Mimi, M. Y. (2018). Effects of horticulture on frail and prefrail nursing home residents: A randomized controlled trial. *Journal of the American Medical Directors Association*, *19*(8), 696-702. | Mean and SD regarding depression were not provided | To examine the effects of HT on the psychosocial well-being of frail and pre-frail nursing home residents |
| 15 | Zhou M. (2015). 园艺治疗提升老年人主观幸福感的方法与实践——以沈阳市养老中心园艺养心园为例［Method and practice of increasing the elderly’s subjective well-Being in horticultural therapy——a case study of mental horticulture garden in Shenyang elderly care center］. In *2015中国园艺疗法研究与实践论文集*(p110-117).北京：中国林业出版社.. | Mean and SD regarding depression were not provided | To find out the effect of horticultural therapy on the psychological health of the elderly. |
| 16 | Huang Q.Y., Kang N., Li X. F., & Li Shuhua.(2020).不同室内园艺活动对老人负性情绪的缓解效益［Effects of different horticultural Activities on the negative emotions of the elderly］. *西北大学学报(自然科学版)，* **50(06)**,887-896. | No report on depression outcomes | To explore whether the horticultural activities have the ability to reduce the negative emotions of the elderly，and to compare the reduction capacity of the two horticultural activities (succulent plants potting and fresh cut flowers arrangement) |
| 17 | Chen C. L., Wu F., Ma Y.M., Wei Y., & Yang R. T. (2020).插花活动对孤寡老人身心健康的影响效应［Study of the effect on empty-nesters' physical and mental health of flower arrangement activity］. *西北大学学报(自然科学版)，***50(06)**,914-922. | No report on depression outcomes | To test the effect on elder people of activities including sensual experience and flower arrangement practice，in which six types of flower materials are selected to the implementation of horticultural therapy on 32 empty-nesters from Beijing Jianwai Warm Home． |
| 18 | Lu H.L. (2018).基于园艺疗法的老年人园艺活动设计及效益评估［Design and benefit evaluation of horticultural activities of the elderly based on horticultural therapy］. *城市建筑*, **17**, 85-87. | No report on depression outcomes | To design a set of standard horticultural therapy team programs |
| 19 | Wei Yu, Dong Zhiyang, Yu Wen, Huang Qiuyun & Li Shuhua.(2020).四种不同园艺疗法活动对孤寡老人的身心健康效益研究［Study on the physical and psychological effect of the four different horticultural activities on the elderly without family members］. *西北大学学报(自然科学版)*, **50(06)**,923-933. | No report on depression outcomes | To investigate the effect of four kinds of horticultural plant cultivation activities，including sowing，transplanting seedlings，succulents potting and herba flower potting, on physical and mental health on the elderly without family members． |
| 20 | Xiu M.L. &Li S.H.(2006). 园艺操作活动对老年人身心健康影响的初步研究［The Effects of Horticultural Operating Activities on Old People’s Physical and Mental Health］. *中国园林*,**06**,46-49. | No report on depression outcomes | To assess the effect of horticultural operating activities on the old people's physical and mental health |
| 21 | Gu W. Y.(2016).园艺疗法在改善老年抑郁症状效果方面的探索［Exploration on the Effect of Horticultural Therapy in Improving the Symptoms of Senile Depression］. *安徽农业科学*, **17**, 272-274. | Mean and SD regarding depression were not provided | To assess the effect of horticultural activities in improving the symptoms of senile depression |

**Supplementary Table 13** Characteristics of HT interventions in the included studies

| **Author and year** | **Country / District** | **Study design** | **Population and sample** | **Tools for outcome assessment** | **Intervention condition and duration** | **Results** |
| --- | --- | --- | --- | --- | --- | --- |
| Edwards *et al.* (2013) | Australia | Quasi-experimental studies | Elderly with dementia recruited from nursing homes  (n=10 for pre- and post- intervention)  Age scope: from 79 to 90 | CSDD | Visiting garden and taking care of the plant; three months | Reduced SCDD scores after HT |
| Yuka (2013) | Japan | Single-blind RCT | Elder women who were residents of the coastal areas of Miyagi Prefecture and had experienced the Great East Japan Earthquake of March 11, 2011and suffering earthquake-related stress  n=39 (HT group =20, Control group =19)  Mean age: HT=65.15 years (SD=3.65) , Control=67.21 years (SD=5.18) | GDS-15 | HT group: 8 weekly sessions (60 minutes each) at a university lab and 15 minutes per day at participants’ homes.  Control group: 8 weekly 60-minute sessions consisting of a lecture regarding stress education. | Reduced GDS-15 scores after HT (both immediate post-effect and follow-up effect) |
| Masuya *et al.* (2014） | Japan | Quasi-experimental studies | Elderly recruited from nursing homes  n=18 (HT group =9, Control group =9)  Mean age: HT group =89.0 years (SD=7.1), Control group =82.2 years (SD=6.6) | GDS-15 | HT group: 6 weekly sessions (30–40 minutes each)  Control Group: 6-week regular care without any intervention | Reduced GDS-15 scores after HT |
| Yuh & Jeng (2015) | Taiwan | Quasi-experimental studies | Elderly recruited from a nursing home (n=10 for pre- and post- intervention)  Mean age=75.3 years (SD=9.55) | GDS-15 | 10 weekly indoor sessions (about 1.5 hours each) | Reduced GDS-15 scores after HT |
| Park *et al*. (2016) | South Korea | Quasi-experimental studies | Elderly women recruited from two senior community centres  n=50 (HT group =24, Control group=26)  Mean age: HT group =79.4 years (SD=4.8), Control group =84.5 years (SD=4.7) | GDSSF-K | HT Group: 15-session intervention conducted for 8 weeks (twice a week, about 50 minutes per session)  Control group: 8-week regular care without any intervention | No significant difference |
| Tik,(2018) | Hong Kong | Quasi-experimental studies | Elderly women with mild depression recruited from an integrated service centre for the elderly  (n=6 for pre- and post- intervention)  Mean age=74.2years | GDS-15 | 6 weekly sessions (60-90 min each) | Reduced GDS-15 scores after HT |
| Hui *et al.* (2019) | Taiwan | RCT | Elderly recruited from three nursing homes  n=150 (HT group =75, Control group =75)  Mean age: HT group =79.2 years (SD=Not available), Control group =77.9 years(SD=Not available) | GDS-15 | HT group: 8 weekly sessions (1.5-2 hours each)  Control group: 8-week regular care without any intervention | Reduced GDS-15 scores after HT |
| Bingying (2020) | China | RCT | Elderly patients hospitalized in geriatrics department  n=90 (HT group =45, Control group =45)  Mean age=Not available | SDS | HT group: 1-2 hours program each time from January 2017 to December 2019.  Control group: Routine daily care without any intervention | Reduced SDS scores after HT |
| Makizako *et al*.(2020) | Japan | Single-blind RCT | Older adults with depressive symptoms and memory problems recruited from one community-based cohort study (conducted since 2011)  Pre-test: n=59 (HT group=30, Control group=29)  Mean age: HT group=73.1 years (SD=5.6), Control group=73.0 years (SD=5.9);  Post-test: n=54 (HT group=26, Control group=28)  12^th^ month follow-up test: n=44 (HT group =20, Control group =24) | GDS-15 | HT Group: 20 weekly sessions (60-90 min each)  Control group: 20-week regular care without any intervention | No significant difference |
| Sia *et al.* (2020) | Singapore | Quasi-experimental studies | Elderly recruited from three senior day care centres  (n=47 for pre- intervention and n=44 for post- intervention)  Mean age: 77.5 years (SD = 7.8) | SDS | 24 weekly sessions in a public garden（60 minutes each)） | No significant difference |
| Yong *et al.* (2020) | South Korea | Quasi-experimental studies | Elderly recruited from a homeless living facility  n=12 (HT group =6, Control group =6)  Mean age: HT group =74 years (SD=5.1) , Control group =72.4 years (SD=5.5) | GDSSF-K | HT group: 16 weekly sessions (60-90 min. each),  Control group: Routine daily life without any intervention | No significant difference |
| Zhen Lan et al (2020) | China | RCT | n=126 (HT group =63, Control group =63)  Mean age: HT group =67.92 years (SD=2.58) , Control group =68.57 years (SD=2.73) | GDS | HT Group: 1-2 hours program each time , six time per week, from March 2018 to March 2019; 30 min. sports nursing program each time, irregularly arranged based on personal willingness , from March 2018 to March 2019  Control group: 30 min. sports nursing program each time, irregularly arranged based on personal willingness , from March 2018 to March 2019 | Reduced GDS scores after HT |
| Mochizuki *et al.* （2021） | Japan | RCT | Older adults with Dementia living in a nursing home  n=16 (HT group =8, Control group =8)  Mean age = 90.3 years (SD=6.8) | CSDD | HT Group： 1 session (average 250 seconds per participant). After that, the participants were asked to decorate their rooms with their floral works for at least 5 days.  Control group: Routine daily care without any intervention | Reduced CSDD scores after HT |

CSDD, Cornell scale for depression in dementia; GDS-15, short version of the Geriatric Depression Scale; GDSSF-K, Korean version of the short form of geriatric depression Scale; HT, horticultural therapy; RCT, randomized controlled trial; SDS, self -rating depression scale;

**Supplementary Table 12** Risk of bias assessment for RCTS

**Supplementary Table 12** Risk of bias assessment for quasi-experimental studies

**Supplementary Figure 1** Forest plot: HT group versus control for depression scores after removing four high risk studies (continuous outcome).


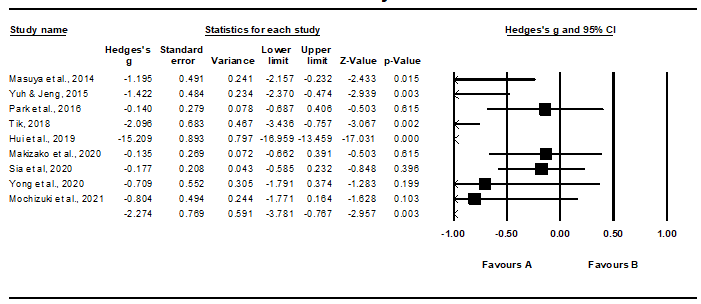


**Supplementary Figure 2** Forest plot: HT group versus control for depression scores after removing two studies where alternative interventions were used in control groups (continuous outcome).


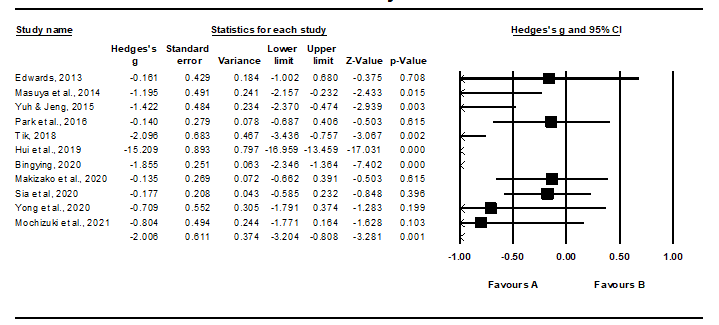

Supplement: Supplementary file 1 [file Data_Sheet_1.docx]
